# Exact ODE Framework for Classical and Quantum Corrections for the Lennard-Jones Second Virial Coefficient †

**DOI:** 10.3390/e27101059

**Published:** 2025-10-11

**Authors:** Zhe Zhao, Alfredo González-Calderón, Jorge Adrián Perera-Burgos, Antonio Estrada, Horacio Hernández-Anguiano, Celia Martínez-Lázaro, Yanmei Li

**Affiliations:** 1 School of Resources and Materials, Northeastern University at Qinhuangdao, Qinhuangdao 066004, China; zhaozhe@neuq.edu.cn; 2 SECIHTI—Department of Geomatic and Hydraulic Engineering, University of Guanajuato, Guanajuato 36000, Gto, Mexico; 3 SECIHTI—Department of Mining, Metallurgy and Geology Engineering, University of Guanajuato, Guanajuato 36020, Gto, Mexico; japerera@secihti.mx; 4 SECIHTI—Centro de Ingeniería y Desarrollo Industrial, Querétaro 76125, Qro, Mexico; jose.estrada@cidesi.edu.mx; 5 Department of Geomatic and Hydraulic Engineering, University of Guanajuato, Guanajuato 36000, Gto, Mexico; horacio.hernandez@ugto.mx; 6 Facultad de Matemáticas, Universidad Autónoma de Guerrero, Chilpancingo 39087, Gro, Mexico; celiamtzlzo@gmail.com; 7 Department of Mining, Metallurgy and Geology Engineering, University of Guanajuato, Guanajuato 36020, Gto, Mexico; yanmeili@ugto.mx

**Keywords:** second virial coefficient, Lennard-Jones fluid, ordinary differential equations (ODEs), Schrödinger-like equation, analytical solutions, quantum correction, special functions

## Abstract

The second virial coefficient (SVC) of the Lennard-Jones fluid is a cornerstone of molecular theory, yet its calculation has traditionally relied on the complex integration of the pair potential. This work introduces a fundamentally different approach by reformulating the problem in terms of ordinary differential equations (ODEs). For the classical component of the SVC, we generalize the confluent hypergeometric and Weber–Hermite equations. For the first quantum correction, we present entirely new ODEs and their corresponding exact-analytical solutions. The most striking result of this framework is the discovery that these ODEs can be transformed into Schrödinger-like equations. The classical term corresponds to a harmonic oscillator, while the quantum correction includes additional inverse-power potential terms. This formulation not only provides a versatile method for expressing the virial coefficient through a linear combination of functions (including Kummer, Weber, and Whittaker functions) but also reveals a profound and previously unknown mathematical structure underlying a classical thermodynamic property.

## 1. Introduction

In 1924, the Lennard-Jones (LJ) potential revolutionized molecular theory by providing a simple, continuous model for pair interactions [1]. As we commemorate its centenary, its history is marked by its pragmatic adoption in the 1920s–30s and its subsequent role as a canonical “Lennard-Jonesium” model, central to early molecular dynamics simulations of liquid argon [2,3]. A recent comprehensive review has reassessed the potential’s enduring achievements and limitations across diverse fields, from intermolecular forces to condensed matter [4]. The original motivation, as J. E. Jones himself articulated, was purely practical: “…No attempt is made to justify this particular attractive field. Its only justification is that it renders the integrals tractable…” [5]. This focus on mathematical tractability has since sparked significant interest in the analytical properties of the LJ fluid, leading to many elegant theoretical results.

A central challenge has been the calculation of the second virial coefficient (SVC), whose integral definition has given rise to a rich but complex landscape of solutions. The classical SVC has been expressed through an infinite series of gamma functions [5,6,7,8,9,10,11,12], as well as in terms of parabolic cylinder functions [13,14,15,16,17,18], Bessel functions [19], and Kummer’s (confluent hypergeometric) functions [8,11,13,17]. A similar diversity exists for the first quantum correction (QC1), which has been formulated using Kummer functions [11] and gamma function series [20,21,22]. While exact, these integration-based methods underscore the inherent mathematical complexity of the problem.

A significant departure from this tradition was pioneered by Eu, who reformulated the classical SVC problem as the solution to an ordinary differential equation (ODE) [13]. This methodological shift from direct integration to differential equations opened a new analytical path. However, this promising ODE-based framework has remained largely confined to the classical component, leaving its full potential unexplored, particularly for quantum effects.

The enduring importance of the SVC is highlighted by a wide range of recent research. Modern studies continue to systematically map $B_{2}\left(T\right)$ across general intermolecular potential families, like the *n*–*m* Lennard-Jones/Mie model [23,24,25], underscoring its role as a critical benchmark. This foundational work directly supports the development of improved reference equations of state for real fluids [26,27,28] and even extends these classical concepts to more abstract theoretical systems, such as hard-hypersphere mixtures in higher dimensions [29]. Furthermore, the fundamental parameters of these potentials, which are often validated against SVC data, prove essential for modeling and understanding modern applied problems, including the temperature-dependent diffusion of gases through nanochannels [30].

On a parallel front, the SVC remains at the heart of high-precision calculations, with ongoing efforts to perform high-accuracy $B_{2}$ evaluations that include ab initio and quantum electrodynamics corrections for light atoms and molecular dimers [31,32]. Beyond simple systems, $B_{2}$ and related parameters are also utilized to capture complex behaviors like fluid anisotropy and quadrupolar effects [33], to construct transferable molecular models for common diatomics like O_2_ and N_2_ [34], and to evaluate quantum corrections for other widely used potentials, such as the Morse potential [35]. Together, these diverse research avenues enrich the context for the present study and confirm the SVC’s multifaceted role in both fundamental theory and modern applications.

In this work, we build upon and significantly extend the ODE-based approach. We present a novel and unified framework of ordinary differential equations that governs both the classical and the first quantum correction (QC1) of the LJ-SVC. Our formulation offers a key advantage: its solutions are directly proportional to the virial components, simplifying their determination. Most strikingly, our framework unveils a previously unknown mathematical structure, revealing that the governing ODEs for the virial components can be transformed into Schrödinger-like equations. The classical term corresponds to a harmonic oscillator, while the quantum correction incorporates additional inverse-power potential terms. This finding, supported by modern analyses of special functions [36,37,38], establishes a profound and unexpected bridge between a classical thermodynamic property and a cornerstone of quantum mechanics.

Motivated by the significant recent advances in machine-learning (ML) interatomic potentials, we include here a brief comment to situate our virial-based ODE formulation for $B_{2}\left(T\right)$ within this context and to outline concrete computational checks. Beyond the analytic ODE formulation, our framework naturally suggests concise numerical validations: (i) low-density molecular dynamics or Monte Carlo (MD/MC), extracting $B_{2}$ from the linear law $Z=1+B_{2}\rho +O\left(\rho^{2}\right)$; (ii) Mayer-sampling Monte Carlo of the exact integral definition of $B_{2}$; (iii) when quantum effects matter, two-particle quantum benchmarks via path-integral Monte Carlo or the Beth–Uhlenbeck formula using scattering phase shifts. Far from being superseded by ML potentials, $B_{2}\left(T\right)$ remains the exact two-body constraint that any force field must satisfy in the dilute limit. The ODE representation provides an efficient, interpretable, and differentiable map $T\mapsto B_{2}\left(T\right)$ that can serve both as a training regularizer enforcing correct low-density behaviour and as a rapid diagnostic of the two-body interaction, while genuine many-body contributions are addressed at the level of higher virial coefficients ($B_{3},B_{4},\ldots$) and finite-density simulations.

This paper is organized as follows. Section 2 lays out the basic formulas for the virial components. Section 3 presents their reformulation as solutions to ODEs, with detailed derivations provided in the Appendix A and Appendix B, and discusses the determination of the integration constants. In Section 4, we explore the physical and mathematical implications of our findings. The paper concludes with a summary of our key results.

## 2. Basic Relationships

The SVC can be expressed as [39]

(1) $$B\left(T\right)=B^{\left(0\right)}\left(T\right)+\frac{\hbar^{2}}{m}B^{\left(1\right)}\left(T\right)+{\left(\frac{\hbar^{2}}{m}\right)}^{2}B^{\left(2\right)}\left(T\right)+\cdots$$

where $B^{\left(0\right)}\left(T\right)$ is the classical term, *T* is the temperature, $B^{\left(1\right)}\left(T\right)$, $B^{\left(2\right)}\left(T\right)$, …, are the first, second, …, quantum corrections to the SVC, *ℏ* is the Planck constant divided by 2π, and *m* is the molecular mass when the fluid is pure. Regarding the reduced LJ potential [1], i.e., $u^{*}\left(x\right)=4\left(x^{-12}-x^{-6}\right)$, *x* being the reduced distance between particles, the classical and the QC1 terms have associated the integrals

(2) $$\begin{array}{cc} B_{c}^{*}\left(\alpha \right) & =-3\int_{0}^{\infty }\left\{e^{-\alpha^{2}u^{*}\left(x\right)}-1\right\}x^{2}dx, \end{array}$$

(3) $$\begin{array}{cc} \frac{B_{q}^{*}\left(\alpha \right)}{24^{2}} & =\alpha^{6}\int_{0}^{\infty }\left\{\frac{4}{x^{24}}-\frac{4}{x^{18}}+\frac{1}{x^{12}}\right\}e^{-\alpha^{2}u^{*}\left(x\right)}dx, \end{array}$$

respectively, where $\alpha^{2}=1/T^{*}$, $T^{*}\equiv k_{B}T/\epsilon$ and $k_{B}$ is the Boltzmann constant, $B_{c}^{*}\equiv B^{\left(0\right)}/\left(2\pi \sigma^{3}/3\right)$, $\Lambda^{*2}B_{q}^{*}\equiv \left(\hbar^{2}/m\right)B^{\left(1\right)}/\left(2\pi \sigma^{3}/3\right)$, $\Lambda^{*2}=\hbar^{2}/\left(4m\sigma^{2}\epsilon \right)$, ϵ is the minimum of energy of the LJ potential, and σ is its collision diameter. It is noted that, by means of the change of variable $t=2x^{-6}$ and integrating by terms, $B_{c}^{*}$ and $B_{q}^{*}$ can be rewritten as follows

(4) $$\begin{array}{cc} B_{c}^{*}\left(\alpha \right) & =-\frac{e^{\alpha^{2}}}{\sqrt{2}}Q_{c}\left(\alpha \right), \end{array}$$

(5) $$\begin{array}{cc} \frac{B_{q}^{*}\left(\alpha \right)}{24} & =2^{\frac{1}{6}}\alpha^{6}e^{\alpha^{2}}Q_{q}\left(\alpha \right), \end{array}$$

where

(6) $$\begin{array}{cc} Q_{c}\left(\alpha \right) & =4\alpha^{2}\left(Q_{-\frac{1}{2}}-Q_{\frac{1}{2}}\right), \end{array}$$

(7) $$\begin{array}{cc} Q_{q}\left(\alpha \right) & =Q_{\frac{5}{6}}-2Q_{\frac{11}{6}}+Q_{\frac{17}{6}}, \end{array}$$

where, in turn,

(8) $$Q_{\chi }\left(\alpha \right)=\int_{0}^{\infty }e^{-\alpha^{2}{\left(t-1\right)}^{2}}t^{\chi }dt.$$

## 3. Formulation of the Virial Terms from ODEs

In this section, we derive the central results of this work: the ordinary differential equations (ODEs) that govern the classical and quantum components of the Lennard-Jones SVC. By starting from the integral representation of the virial components, $Q_{c}$ and $Q_{q}$, and applying a series of differentiations and recurrence relations (with detailed derivations provided in the Appendix A and Appendix B), we obtain the governing ODEs. This framework allows the virial components to be expressed elegantly as a linear combination of two independent solutions, whose coefficients can then be determined from experimental data or asymptotic limits.

### 3.1. Classical Term of the Virial

Our first goal is to derive the governing ODE for the classical component of the SVC, which is proportional to $Q_{c}\left(\alpha \right)$. Following the mathematical procedure detailed in Appendix A—which involves differentiation of the integral definition of $Q_{\chi }$ and the use of recurrence relations—we arrive at the following second-order differential equation:

(9) $$\frac{d^{2}Q_{c}}{d\alpha^{2}}+\left(2\alpha -\frac{1}{\alpha }\right)\frac{dQ_{c}}{d\alpha }+\left(2+\frac{3}{4\alpha^{2}}\right)Q_{c}=0,$$

Equation (9) is a generalization of the confluent hypergeometric equation [40]. While it provides a complete description, a change of variable, $\psi \to \alpha^{-\frac{1}{2}}e^{\frac{1}{2}\alpha^{2}}Q_{c}$, followed by a rescaling of the independent variable, $\alpha \to \alpha /\sqrt{2}$, transforms it into a more familiar and fundamental form:

(10) $$\begin{array}{cc} \frac{d^{2}\psi }{d\alpha^{2}}+\left(1-\frac{1}{4}\alpha^{2}\right)\psi & =0. \end{array}$$

This is the canonical Weber–Hermite parabolic cylinder equation [41], which is closely related to the quantum harmonic oscillator. Given that its solutions are well-known, the classical virial coefficient $B_{c}^{*}\left(\alpha \right)=-e^{\alpha^{2}}Q_{c}\left(\alpha \right)/\sqrt{2}$ can be expressed as a linear combination of two independent solutions, either in terms of confluent hypergeometric (Kummer’s) functions or parabolic cylinder (Weber’s) functions:

(11) Bc*(T*)=−T*−142{c1F11−14,12,1T*+c2U−14,12,1T*,e12T*c1D122T*+c2D12−2T*,

where F11(a,b,c) and U(a,b,c) are Kummer’s functions, of first and second kind, respectively. $D_{a}\left(\pm b\right)$ are Weber functions, and $c_{1}$ and $c_{2}$ are constants to be determined by solving the two-point boundary values problem.

### 3.2. QC1 Term of the Virial

Having established the ODE framework for the classical virial coefficient, we now turn to the significantly more complex first quantum correction (QC1), represented by the term $Q_{q}\left(\alpha \right)$. Following a procedure analogous to the classical case—detailed in Appendix B—we derive the governing differential equation. The process involves a more intricate algebraic path, leading to a system of coupled equations that, when solved, yields a novel second-order ODE for $Q_{q}$:

(12) $$\begin{array}{cc} \frac{d^{2}Q_{q}}{d\alpha^{2}} & +\frac{432\alpha^{4}+1554\alpha^{2}+1265}{3\alpha \left(72\alpha^{2}+55\right)}\frac{dQ_{q}}{d\alpha }\\ \; & +\frac{15552\alpha^{4}+23760\alpha^{2}+21505}{36\alpha^{2}\left(72\alpha^{2}+55\right)}Q_{q}=0, \end{array}$$

To the best of our knowledge, this ordinary differential equation has not been reported in the literature previously. Just as in the classical case, this complex equation can be transformed into a more physically insightful form. By applying the transformation $\psi \equiv \alpha^{\frac{23}{6}}e^{\frac{1}{2}\alpha^{2}}{\left(72\alpha^{2}+55\right)}^{-\frac{1}{2}}Q_{q}$, we obtain a Schrödinger-like equation:

(13) $$\begin{array}{cc} \frac{d^{2}\psi }{d\alpha^{2}} & -\left(\frac{2}{3}+\alpha^{2}+\frac{154}{72\alpha^{2}+55}-\frac{11880}{{\left(72\alpha^{2}+55\right)}^{2}}\right)\psi =0, \end{array}$$

This equation describes a harmonic oscillator subjected to additional inverse-power potential terms, revealing a deep structural parallel with its classical counterpart.

Unlike the well-documented Weber–Hermite equation, the solutions to Equations (12) and (13) are not standard and cannot be readily found. However, an exact closed-form solution can be constructed by leveraging the known integral solution for the QC1 term, as given by Michels [11]. In order to bridge our ODE with the physical virial coefficient, we first recall their relationship:

(14) $$B_{q}^{*}\left(\alpha \right)=24\cdot 2^{\frac{1}{6}}\alpha^{6}e^{\alpha^{2}}Q_{q}\left(\alpha \right).$$

By identifying the functions that constitute Michels’s result, we can deduce the two linearly independent solutions, $y_{1},y_{2}$ and $Y_{1},Y_{2}$, for our new ODEs. This allows the QC1 term of the LJ-SVC to be written as:

(15) $$B_{q}^{*}\left(T^{*}\right)=24\cdot 2^{1/6}\{\begin{array}{c} {T^{*}}^{-3}e^{\frac{1}{T^{*}}}\left[c_{1}y_{1}\left(T^{*}\right)+c_{2}y_{2}\left(T^{*}\right)\right],\\ \begin{array}{c} {T^{*}}^{-\frac{13}{12}}e^{\frac{1}{2T^{*}}}{\left(72{T^{*}}^{-1}+55\right)}^{\frac{1}{2}}\times \left[c_{1}Y_{1}\left(T^{*}\right)+c_{2}Y_{2}\left(T^{*}\right)\right], \end{array} \end{array}$$

where $y_{1}$ and $y_{2}$ are the solutions to Equation (12), and $Y_{1}$ and $Y_{2}$ are the solutions to the Schrödinger-like Equation (13). These solutions are explicitly constructed from two basis functions, $F(T^{*})$ and $G(T^{*})$, as follows:

(16) $$\begin{array}{cc} y_{1}\left(T^{*}\right) & ={T^{*}}^{3}e^{-\frac{1}{T^{*}}}F\left(T^{*}\right), \end{array}$$

(17) $$\begin{array}{cc} y_{2}\left(T^{*}\right) & ={T^{*}}^{3}e^{-\frac{1}{T^{*}}}G\left(T^{*}\right), \end{array}$$

(18) $$\begin{array}{cc} Y_{1}\left(T^{*}\right) & =\frac{{T^{*}}^{\frac{13}{12}}e^{-\frac{1}{2T^{*}}}F\left(T^{*}\right)}{{\left(72/T^{*}+55\right)}^{\frac{1}{2}}}, \end{array}$$

(19) $$\begin{array}{cc} Y_{2}\left(T^{*}\right) & =\frac{{T^{*}}^{\frac{13}{12}}e^{-\frac{1}{2T^{*}}}G\left(T^{*}\right)}{{\left(72/T^{*}+55\right)}^{\frac{1}{2}}}. \end{array}$$

The basis functions $F(T^{*})$ and $G(T^{*})$ are, in turn, composed of confluent hypergeometric functions:

(20) F(T*)=1T*191272F11512;12;1T*−22F11512;32;1T*,

(21) G(T*)=1T*131212T*F111112;32;1T*+11F11−112;12;1T*.

This result provides the first-ever exact solution to the newly discovered differential equations governing the QC1 of the Lennard-Jones second virial coefficient, with the constants $c_{1}$ and $c_{2}$ to be determined from boundary conditions.

### 3.3. Values of the Constants in the Linear Combination

The constants of integration, $c_{1}$ and $c_{2}$, determine the specific physical solution from the general mathematical framework. They can be determined using two primary methods: by solving a two-point boundary value problem or by matching the solution to its known asymptotic behavior at extreme temperatures.

#### 3.3.1. Method 1: Two-Point Boundary Value Problem

The most direct way to determine the constants is by fitting the general solution to two known data points. Let $b_{1}$ and $b_{2}$ be the known values of the virial coefficient ($B_{c}^{*}$ or $B_{q}^{*}$) at two distinct reduced temperatures, $T_{1}^{*}$ and $T_{2}^{*}$. This sets up a system of two linear equations:

(22) $$\begin{array}{c} b_{1}=c_{1}\gamma \left(T_{1}^{*}\right)+c_{2}\delta \left(T_{1}^{*}\right) \end{array}$$

(23) $$\begin{array}{c} b_{2}=c_{1}\gamma \left(T_{2}^{*}\right)+c_{2}\delta \left(T_{2}^{*}\right) \end{array}$$

where $\gamma (T^{*})$ and $\delta (T^{*})$ represent the corresponding linearly independent basis functions from Equation (11) or (15). Solving this system yields the unique constants:

(24) $$\begin{array}{cc} c_{1} & =\frac{b_{1}\delta \left(T_{2}^{*}\right)-b_{2}\delta \left(T_{1}^{*}\right)}{\gamma \left(T_{1}^{*}\right)\delta \left(T_{2}^{*}\right)-\gamma \left(T_{2}^{*}\right)\delta \left(T_{1}^{*}\right)},\\ c_{2} & =\frac{b_{2}\gamma \left(T_{1}^{*}\right)-b_{1}\gamma \left(T_{2}^{*}\right)}{\gamma \left(T_{1}^{*}\right)\delta \left(T_{2}^{*}\right)-\gamma \left(T_{2}^{*}\right)\delta \left(T_{1}^{*}\right)}. \end{array}$$

To validate our framework, we can use the well-established exact-analytical solutions for the virial components as the reference data points. For the classical term, this is [16,17,18]:

(25) $$B_{c}^{*}\left(T^{*}\right)=\sqrt{2\pi }{T^{*}}^{-\frac{1}{4}}e^{\frac{1}{2T^{*}}}D_{\frac{1}{2}}\left(-\sqrt{\frac{2}{T^{*}}}\right),$$

and for the first quantum correction [11]:

(26) $$\begin{array}{c} \frac{B_{q}^{*}\left(T^{*}\right)}{\left(2^{1/6}/12\right)}=\Gamma \left(\frac{5}{12}\right)F\left(T^{*}\right)-\Gamma \left(-\frac{1}{12}\right)G\left(T^{*}\right). \end{array}$$

**Figure 1:**
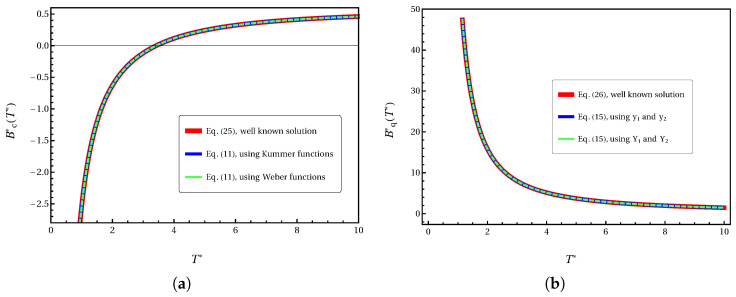
Validation of the ODE framework for the (**a**) classical second virial coefficient, $B_{c}^{*}\left(T^{*}\right)$, and (**b**) the first quantum correction, $B_{q}^{*}\left(T^{*}\right)$. The solid red line represents the well-known exact solution (Equations (25) and (26)). The square and dashed-line markers represent the solutions obtained from our ODEs (Equations (11) and (15)), with the integration constants determined by the two-point boundary value method at $T_{1}^{*}=1.0$ and $T_{2}^{*}=10.0$. The perfect overlap demonstrates the exactness of our formulation.

Figure 1 demonstrates the robustness of our method. By using the values from Equations (25) and (26) at $T_{1}^{*}=1.0$ and $T_{2}^{*}=10.0$ as boundary conditions, our formulation perfectly reproduces these exact solutions across the entire temperature range, as shown by the overlapping curves.

#### 3.3.2. Method 2: Asymptotic Series Expansion

An alternative method to determine the constants involves comparing the series expansion of our general solutions with their known asymptotic forms in the limits of very high ($T^{*}\to \infty$) or very low ($T^{*}\to 0$) temperatures. The established asymptotic behaviors for the classical term [18] are as follows:

(27) $$B_{c}^{*}\left(T^{*}\right)=\left\{\begin{array}{c} \sqrt{2}\left[\frac{\Gamma \left(\frac{3}{4}\right)}{{T^{*}}^{\frac{1}{4}}}-\frac{1}{2}\frac{\Gamma \left(\frac{1}{4}\right)}{{T^{*}}^{\frac{3}{4}}}+\cdots \right],\\ -\sqrt{\frac{\pi }{2}}e^{\frac{1}{T^{*}}}\left[{T^{*}}^{\frac{1}{2}}+\frac{15}{16}{T^{*}}^{\frac{3}{2}}+\cdots \right]. \end{array}\right.$$

In this equation, the first equality is for $T^{*}\to \infty$ and the second equality is for $T^{*}\to 0$. Similarly, for the QC1 term, the known behaviors are as follows [11,20]:

(28) $$\;\frac{B_{q}^{*}\left(T^{*}\right)}{2^{\frac{1}{6}}}=\left\{\begin{array}{c} -\frac{11\Gamma \left(-\frac{1}{12}\right)}{12{T^{*}}^{\frac{13}{12}}}+\frac{25\Gamma \left(\frac{5}{12}\right)}{6{T^{*}}^{\frac{13}{12}}}\frac{1}{{T^{*}}^{\frac{1}{2}}}+\cdots ,\\ \frac{1}{\sqrt{\pi^{3}}}\frac{e^{\frac{1}{T^{*}}}}{{T^{*}}^{\frac{1}{2}}}\left[-\frac{11}{96}+\frac{3}{4}\frac{1}{T^{*}}+\cdots \right]. \end{array}\right.$$

By expanding our general solutions from Equations (11) and (15) and matching the coefficients term by term, we obtain the values for $c_{1}$ and $c_{2}$ summarized in Table 1. The details of these expansions are as follows.

##### Classical Virial Coefficient ($B_{c}^{*}$)

In the high-temperature limit ($T^{*}\to \infty$), where $z=1/T^{*}\to 0$, the confluent hypergeometric functions from Equation (11) expand as follows:

F11−14,12,1T*=1−12T*+O(T*−2),U−14,12,1T*=Γ(1/2)Γ(1/4)+O(T*−1/2).

In the low-temperature limit ($T^{*}\to 0$), the asymptotic forms of the parabolic cylinder functions dominate.

##### Quantum Virial Coefficient ($B_{q}^{*}$)

For the QC1 term in the high-temperature limit, expanding the basis functions $F(T^{*})$ and $G(T^{*})$ yields:

$$\begin{array}{cc} F(T^{*}) & =50{T^{*}}^{-\frac{19}{12}}+O\left({T^{*}}^{-\frac{31}{12}}\right),\\ G(T^{*}) & =11{T^{*}}^{-\frac{13}{12}}+O\left({T^{*}}^{-\frac{25}{12}}\right). \end{array}$$

In the low-temperature limit, both $F(T^{*})$ and $G(T^{*})$ are found to be dominated by an $e^{1/T^{*}}$ term.

The results of this matching procedure, presented in Table 1, are revealing. In the high-temperature limit ($T^{*}\to \infty$), the method is robust and the coefficients correctly reproduce the well-known formulas obtained from direct integration.

In contrast, the low-temperature limit ($T^{*}\to 0$) requires careful consideration. For the QC1 term, the asymptotic forms of the basis functions $F(T^{*})$ and $G(T^{*})$ share a dominant exponential term $e^{1/T^{*}}$. Attempting to determine two constants by matching the sub-dominant algebraic terms leads to an ill-conditioned problem, resulting in the “indefinite” coefficients. This does not imply the solution is invalid, but rather that the asymptotic matching method is not a stable procedure for uniquely fixing both constants in this limit. This highlights the general superiority of the two-point boundary value method for ensuring global accuracy.

## 4. Discussion: Mathematical Structure and Physical Implications

The formulation of the second virial coefficient in terms of ordinary differential equations, as presented in this work, yields several profound insights that extend beyond providing an alternative computational method. Here, we discuss the interconnectedness of the solutions, the physical meaning of the emergent Schrödinger-like structure, and the potential universality of this phenomenon.

### 4.1. The Rich Interconnectedness of the Analytical Solutions

A direct consequence of our ODE framework is the revelation that the classical SVC can be expressed through a variety of special functions. The equivalences between confluent hypergeometric functions (F11, *U*), parabolic cylinder functions ($D_{\nu }$), Whittaker functions ($M_{\kappa ,\mu }$, $W_{\kappa ,\mu }$), and Hermite polynomials ($He_{\nu }$) are well-known mathematical identities [40]. A direct consequence of our ODE framework is the revelation that the classical SVC can be expressed through a variety of special functions. This is not merely a matter of convenience; it points to a deeply interconnected mathematical space where the solution resides. For instance, the Whittaker functions ($M_{\kappa ,\mu }$, $W_{\kappa ,\mu }$), which solve Whittaker’s differential equation, are themselves defined directly in terms of the confluent hypergeometric (Kummer’s) functions that appear in our primary solutions [40]:

(29) Mκ,μ(z)=e−z/2zμ+1/2F11(μ−κ+1/2,2μ+1,z),

(30) $$\begin{array}{cc} W_{\kappa ,\mu }\left(z\right) & =e^{-z/2}z^{\mu +1/2}U\left(\mu -\kappa +1/2,2\mu +1,z\right). \end{array}$$

This interconnectedness is vividly illustrated by the specific Kummer U-function that forms one of the basis solutions for the classical SVC. It admits a remarkable set of equivalent representations spanning different families of special functions:

(31) $$U\left(-\frac{1}{4},\frac{1}{2},x^{2}\right)=\{\begin{array}{c} x\;U\left(\frac{1}{4},\frac{3}{2},x^{2}\right)\\ 2^{-\frac{1}{4}}e^{\frac{1}{4}x^{2}}D_{\frac{1}{2}}\left(x\right)\\ 2^{-\frac{1}{2}}x^{\frac{1}{2}}W_{\frac{1}{2},-\frac{1}{4}}\left(\frac{1}{2}x^{2}\right)\\ 2^{-\frac{1}{2}}He_{\frac{1}{2}}\left(x\right) \end{array}$$

Similarly, its counterpart, the Kummer F-function, also possesses multiple equivalent forms that connect it to the Whittaker and parabolic cylinder functions:

(32) F11−14,12,x={ex/2x−1/4M12,−14(x)2−1/4ex/2D122x

Within this context, these identities are not mere mathematical curiosities. They demonstrate that the solution to the virial problem resides in a deeply interconnected mathematical space. Our ODE approach acts as a unifying framework from which any of these equivalent representations can be systematically derived, offering significant analytical flexibility.

### 4.2. The Emergent Schrödinger-like Structure

Perhaps the most significant finding of this work is the transformation of the governing ODEs for both the classical and QC1 components into the canonical form of a one-dimensional, time-independent Schrödinger equation (Equations (10) and (13)).

(33) $$-\frac{d^{2}\psi }{d\alpha^{2}}+V\left(\alpha \right)\psi =E\psi$$

For the classical term, the equation is precisely that of a quantum harmonic oscillator with $V\left(\alpha \right)\propto \alpha^{2}$ and a fixed energy eigenvalue. For the QC1 term, the effective potential V(α) acquires additional terms resembling repulsive dipole and higher-order inverse-power potentials. This emergent structure is remarkable: it implies that the thermodynamic properties of a classical fluid, when viewed through the lens of its temperature dependence ($\alpha^{2}=1/T^{*}$), are governed by a mathematical formalism identical to that which governs the stationary states of a quantum particle.

### 4.3. Evidence for Universality: The Square-Well Fluid

The question arises whether this Schrödinger-like structure is a unique artifact of the Lennard-Jones potential’s functional form. Evidence suggests it is not. The classical SVC for the analytically simpler square-well (SW) potential also yields a Schrödinger-like equation. By applying the transformation $\psi \equiv \alpha^{-\frac{1}{2}}e^{-\frac{1}{2}\alpha^{2}}B_{SW}^{*}$, where $B_{SW}^{*}$ is the reduced SW virial coefficient, one obtains the following equation [42]:

(34) $$\frac{d^{2}\psi }{d\alpha^{2}}-\left(\frac{3}{4\alpha^{2}}+\alpha^{2}\right)\psi =0,$$

This equation corresponds to a harmonic oscillator in a repulsive dipole potential ($1/\alpha^{2}$) with a zero-energy eigenvalue. The fact that this structure appears for at least two fundamentally different potentials (a continuous one and a discontinuous one) strongly suggests that the connection between the SVC and second-order linear differential equations of this form may be a more general, perhaps universal, feature of the two-body problem in statistical mechanics. Our work places these findings within a unified and rigorous framework.

## 5. Conclusions

In this work, we have presented a fundamental shift in the analytical treatment of the second virial coefficient (SVC) for the Lennard-Jones fluid, moving from traditional integral-based methods to a more powerful framework based on ordinary differential equations (ODEs). This approach not only provides new, flexible methods for computation but also uncovers a previously hidden mathematical structure underlying a classical thermodynamic property.

Our primary contribution is the derivation of novel, exact ODEs for both the classical component and the first quantum correction (QC1) of the LJ-SVC. To the best of our knowledge, the differential equations presented for the QC1 term, Equations (12) and (13), are new to the literature. A key achievement of this work is the discovery of their exact, closed-form solutions, which we constructed by leveraging a known integral result [11]. These solutions can be expressed as a linear combination of well-known special functions, and we have established robust methods for determining their physical constants.

The most profound outcome of this study is the discovery that the governing equations for the virial components can be transformed into one-dimensional, time-independent Schrödinger-like equations. The classical SVC is shown to be mathematically equivalent to the problem of a quantum harmonic oscillator, while the QC1 term corresponds to a harmonic oscillator perturbed by inverse-power potentials. The corroborating evidence from the square-well fluid suggests this is not an isolated coincidence but may be a general feature of the second virial coefficient.

This emergent Schrödinger structure implies a deep isomorphism between the statistical mechanics of interacting particles and the formalism of quantum mechanics, opening a new theoretical avenue for exploring the mathematical foundations of thermodynamics. Future work should focus on investigating whether similar structures govern higher-order virial coefficients or the thermodynamic properties of other, more complex potentials. The framework developed herein provides the conceptual foundation for such an endeavor.

## Figures and Tables

**Table 1:** Coefficient values for the Equations (11) and (15) which are obtained at very low or very high temperature.

	$T^{*}\to 0$ ^a^		$T^{*}\to \infty$	
From Equation	$c_{1}$	$c_{2}$	$c_{1}$	$c_{2}$
(9)	$-4\;\Gamma \left(\frac{3}{4}\right)$	0	$-4\;\Gamma \left(\frac{3}{4}\right)$	$2\sqrt{2\pi }$
(10)	0	$-2\cdot 2^{\frac{1}{4}}\sqrt{\pi }$	0	$-2\cdot 2^{\frac{1}{4}}\sqrt{\pi }$
(12) and (13)	indefinite	indefinite	$\frac{1}{288}\Gamma \left(\frac{5}{12}\right)$	$-\frac{1}{288}\Gamma \left(-\frac{1}{12}\right)$

^a^ The coefficients from Equation (9) give rise to a correct behavior of the virial only at low temperature. Meanwhile, coefficients from Equation (10) allow obtain a correct virial in the whole range of temperature. However, indefinite coefficients are obtained for the first quantum virial correction of the virial.

## Data Availability

No new data were created or analyzed in this study. Data sharing is not applicable to this article.

## Abbreviations

The following abbreviations are used in this manuscript:

LJ	Lennard-Jones
ODE	Ordinary Differential Equation
SVC	Second Virial Coefficient

## Appendix A. Derivation of the ODE for the Classical Approximation

This appendix derives the ODE satisfied by the classical contribution to the Lennard-Jones second virial coefficient (LJ–SVC). The reduced classical LJ integral is recalled (after introducing reduced variables x≡r/σ, $T^{*}\equiv k_{B}T/\varepsilon$, and $\alpha \equiv 1/\sqrt{T^{*}}$):

$$I\left(\alpha \right)\;=\;\int_{0}^{\infty }\;\left(e^{-4\alpha^{2}\left(x^{-12}-x^{-6}\right)}-1\right)\;x^{2}\;dx.$$

The change of variables $t=2x^{-6}$ (so that $t/2=x^{-6}$, $x^{-1}dx=-\left(1/6\right)\;t^{-1}dt$, hence $x^{2}dx=-\left(\sqrt{2}/6\right)\;t^{-3/2}dt$) yields

$$I\left(\alpha \right)\;=\;\frac{\sqrt{2}}{6}\int_{0}^{\infty }\;\left(e^{-\alpha^{2}\left(t^{2}-2t\right)}-1\right)t^{-3/2}dt.$$

Using $t^{2}-2t={\left(t-1\right)}^{2}-1$, we factor out the constant piece inside the exponential,

$$I\left(\alpha \right)\;=\;\frac{\sqrt{2}}{6}\;e^{\alpha^{2}}\;\int_{0}^{\infty }\;\left(e^{-\alpha^{2}{\left(t-1\right)}^{2}}-e^{-\alpha^{2}}\right)t^{-3/2}dt\;\equiv \;\frac{\sqrt{2}}{6}\;e^{\alpha^{2}}J\left(\alpha \right).$$

Integrating J(α) by parts with

$$u=e^{-\alpha^{2}{\left(t-1\right)}^{2}}-e^{-\alpha^{2}}\;and\;dv=t^{-3/2}dt,$$

the boundary term vanishes (the Gaussian controls the t→∞ endpoint and the square bracket cancels at $t\to 0^{+}$), and we obtain

$$J\left(\alpha \right)=-4\alpha^{2}\int_{0}^{\infty }\;\left(t-1\right)\;e^{-\alpha^{2}{\left(t-1\right)}^{2}}t^{-1/2}dt=-4\alpha^{2}\;\left(\int_{0}^{\infty }\;e^{-\alpha^{2}{\left(t-1\right)}^{2}}t^{1/2}dt-\int_{0}^{\infty }\;e^{-\alpha^{2}{\left(t-1\right)}^{2}}t^{-1/2}dt\right).$$

This motivates working with the Gaussian-weighted moments

(A1) $$Q_{\pm \frac{1}{2}}\left(\alpha \right)=\int_{0}^{\infty }e^{-\alpha^{2}{\left(t-1\right)}^{2}}\;t^{\pm \frac{1}{2}}\;dt,$$

and with the finite α-scaled difference

$$Q_{c}\;\equiv \;4\alpha^{2}\;\left(Q_{-\frac{1}{2}}-Q_{\frac{1}{2}}\right),$$

for which one has simply $J\left(\alpha \right)=Q_{c}\left(\alpha \right)$ and consequently $I\left(\alpha \right)=\left(\sqrt{2}/6\right)e^{\alpha^{2}}Q_{c}\left(\alpha \right)$. Thus $Q_{c}$ captures the entire classical LJ integral up to an overall, physically irrelevant prefactor. The combination $Q_{c}$ is also algebraically convenient: α–derivatives and the by–parts recurrences below close on the pair $\left\{Q_{-1/2},Q_{1/2}\right\}$ and therefore on $Q_{c}$ itself.

Integrating by parts the family $Q_{-k/2}$ with

$$u=e^{-\alpha^{2}{\left(t-1\right)}^{2}},\;dv=t^{-k/2}\;dt,$$

so that $du=-2\alpha^{2}\left(t-1\right)e^{-\alpha^{2}{\left(t-1\right)}^{2}}dt$ and $v=\frac{2}{2-k}\;t^{\;1-k/2}$, the boundary term $uv{|}_{0}^{\infty }$ vanishes for integer k<2 (the Gaussian controls t→∞ and $t^{1-k/2}$ is integrable at 0). We obtain

$$\begin{array}{cc} Q_{-\frac{k}{2}}= & \frac{2}{2-k}t^{1-\frac{k}{2}}e^{-\alpha^{2}{\left(t-1\right)}^{2}}{|}_{0}^{\infty }\\ \; & +\frac{4\alpha^{2}}{2-k}\int_{0}^{\infty }\left(t-1\right)t^{1-\frac{k}{2}}e^{-\alpha^{2}{\left(t-1\right)}^{2}}\;dt, \end{array}$$

which is the recurrence quoted as

(A2) $$Q_{-\frac{k}{2}}=\frac{4\alpha^{2}}{2-k}\;\left(Q_{2-\frac{k}{2}}-Q_{1-\frac{k}{2}}\right).$$

Differentiating (A1) with respect to α (under the integral sign) gives, for any exponent ξ,

$$\frac{dQ_{\xi }}{d\alpha }=-2\alpha \;\int_{0}^{\infty }e^{-\alpha^{2}{\left(t-1\right)}^{2}}\left(t^{2}-2t+1\right)t^{\xi }\;dt=-2\alpha \left(Q_{\xi +2}-2Q_{\xi +1}+Q_{\xi }\right).$$

Using the recurrence (A2) to reduce $Q_{\xi +1}$ and $Q_{\xi +2}$ back to $\xi =\pm \frac{1}{2}$ one closes the system on $\left\{Q_{-1/2},Q_{1/2}\right\}$. After a short algebra, this yields

(A3) $$\begin{array}{ccc} 8\alpha^{3}\;\frac{dQ_{\frac{1}{2}}}{d\alpha } & = & Q_{c}-8\alpha^{2}Q_{\frac{1}{2}},\\ 8\alpha^{3}\;\frac{dQ_{-\frac{1}{2}}}{d\alpha } & = & -4\alpha^{2}Q_{-\frac{1}{2}}-4\alpha^{2}Q_{c}. \end{array}$$

Subtracting the two lines in (A3) and writing the result as the derivative of $Q_{c}/\left(4\alpha^{2}\right)$ one finds

$$8\alpha^{3}\frac{d\left[Q_{c}/\left(4\alpha^{2}\right)\right]}{d\alpha }=-\left(4\alpha^{2}+2\right)Q_{c}+4\alpha^{2}Q_{\frac{1}{2}},$$

which, upon differentiating the left-hand side and simplifying, gives the convenient first-order relation

(A4) $$\alpha \frac{dQ_{c}}{d\alpha }=2\alpha^{2}Q_{\frac{1}{2}}+\left(1-2\alpha^{2}\right)Q_{c}.$$

Differentiating (A4) with respect to α and eliminating $Q_{\frac{1}{2}}$ and its derivative by means of (A3) yields, after straightforward algebra,

(A5) $$\frac{d^{2}Q_{c}}{d\alpha^{2}}+\left(2\alpha -\frac{1}{\alpha }\right)\frac{dQ_{c}}{d\alpha }+\left(2+\frac{3}{4\alpha^{2}}\right)Q_{c}=0.$$

A standard exponential integrating factor

(A6) $$\mathrm{Exp}\;\left[\frac{1}{2}\int \frac{Q(\alpha )}{P(\alpha )}\;d\alpha \right],$$

with P(α)=1 and Q(α)=2α−1/α in (A5), leads to

$$\psi \left(\alpha \right)\;\equiv \;\alpha^{-\frac{1}{2}}\;e^{\alpha^{2}/2}\;Q_{c}\left(\alpha \right),$$

which satisfies the Schrödinger-type equation

$$\frac{d^{2}\psi }{d\alpha^{2}}+\left(2-\alpha^{2}\right)\psi =0.$$

Finally, with the scaling $\alpha^{\prime }=\alpha /\sqrt{2}$, we reach the Weber–Hermite (parabolic cylinder) form

$$\frac{d^{2}\psi }{d{\alpha^{\prime }}^{2}}+\left(1-\frac{1}{4}{\alpha^{\prime }}^{2}\right)\psi =0.$$

## Appendix B. Derivation of the ODE for the First Quantum Correction

This appendix derives the ordinary differential equation (ODE) governing the first quantum correction (QC1) of the Lennard-Jones second virial coefficient (LJ–SVC). In the QC1 contribution to the second virial coefficient one encounters integrals of the form

$$\int_{0}^{\infty }{\left(-\frac{d}{dr}u\left(r\right)\right)}^{2}e^{-u\left(r\right)/\left(k_{B}T\right)}r^{2}\;dr,$$

which, after changing to reduced variables, for the Lennard-Jones 12–6 potential, x≡r/σ and $\alpha \equiv 1/\sqrt{T^{*}}$, may be mapped to Gaussian-weighted moments by the substitution $t=2x^{-6}$ (so that $e^{-4\alpha^{2}\left(x^{-12}-x^{-6}\right)}=e^{-\alpha^{2}\left[{\left(t-1\right)}^{2}-1\right]}$ and $x^{-1}dx=-\left(2^{1/6}/6\right)t^{-7/6}dt$). This leads naturally to the family

(A7) $$Q_{\frac{k}{6}}\left(\alpha \right)=\int_{0}^{\infty }e^{-\alpha^{2}{\left(t-1\right)}^{2}}\;t^{\frac{k}{6}}\;dt,$$

and to the specific QC1 linear combination

$$Q_{q}\equiv Q_{\frac{5}{6}}-2Q_{\frac{11}{6}}+Q_{\frac{17}{6}},$$

which is directly proportional to the reduced quantum contribution $B_{q}^{*}\left(T^{*}\right)$ in the notation of the main text.

Integrating (A7) by parts with $u=e^{-\alpha^{2}{\left(t-1\right)}^{2}}$ and $dv=t^{k/6}\;dt$, the boundary term $uv{|}_{0}^{\infty }$ vanishes for k>−6 (the Gaussian controls the t→∞ endpoint and $t^{1+k/6}$ is integrable at 0). We then find the convergent relation

(A8) $$\begin{array}{cc} Q_{\frac{k}{6}}\; & =\frac{6}{k+6}\;t^{1+\frac{k}{6}}e^{-\alpha^{2}{\left(t-1\right)}^{2}}{|}_{0}^{\infty }+\frac{12\alpha^{2}}{k+6}\int_{0}^{\infty }e^{-\alpha^{2}{\left(t-1\right)}^{2}}\;\left(t-1\right)\;t^{1+\frac{k}{6}}\;dt, \end{array}$$

which implies the recurrence

(A9) $$Q_{\frac{k}{6}}=\frac{12\alpha^{2}}{k+6}\left(Q_{\frac{k+12}{6}}-Q_{\frac{k+6}{6}}\right).$$

Differentiating (A7) with respect to α (under the integral sign) and using (A9) to remove higher moments gives the closed first-derivative system

(A10) $$\begin{array}{ccc} 6\alpha \;\frac{dQ_{\frac{5}{6}}}{d\alpha } & = & 12\alpha^{2}\left(Q_{\frac{11}{6}}-Q_{\frac{5}{6}}\right)-11Q_{\frac{5}{6}},\\ 6\alpha \;\frac{dQ_{\frac{11}{6}}}{d\alpha } & = & 11Q_{\frac{5}{6}}-17Q_{\frac{11}{6}},\\ 6\alpha \;\frac{dQ_{\frac{17}{6}}}{d\alpha } & = & 17Q_{\frac{11}{6}}-23Q_{\frac{17}{6}}. \end{array}$$

A direct use of (A9) with k=5 gives

(A11) $$Q_{\frac{5}{6}}=\frac{12}{11}\alpha^{2}\left(Q_{\frac{17}{6}}-Q_{\frac{11}{6}}\right),$$

so that, in particular,

(A12) $$Q_{q}=\left(\frac{12}{11}\alpha^{2}+1\right)Q_{\frac{17}{6}}-\left(\frac{12}{11}\alpha^{2}+2\right)Q_{\frac{11}{6}}.$$

From (A12) we obtain, after one differentiation and the replacement of $Q_{5/6}$ via (A11),

(A13) $$\frac{dQ_{q}}{d\alpha }=\frac{2}{\alpha }Q_{\frac{5}{6}}+\left(\frac{12}{11}\alpha^{2}+1\right)\frac{d}{d\alpha }\left(\frac{11}{12\alpha^{2}}Q_{\frac{5}{6}}\right)-\frac{dQ_{\frac{11}{6}}}{d\alpha }.$$

To make the link between $Q_{q}$ and the derivative of $Q_{5/6}$ explicit, start from the recurrence consequence (A11) and differentiate it with respect to α:

(A14) $$\frac{dQ_{\frac{5}{6}}}{d\alpha }=\frac{24}{11}\;\alpha \left(Q_{\frac{17}{6}}-Q_{\frac{11}{6}}\right)+\frac{12}{11}\;\alpha^{2}\;\left(\frac{dQ_{\frac{17}{6}}}{d\alpha }-\frac{dQ_{\frac{11}{6}}}{d\alpha }\right).$$

Next, eliminate the derivatives on the right-hand side using the second and third relations in (A10), to rewrite the derivative difference as

(A15) $$\frac{dQ_{\frac{17}{6}}}{d\alpha }-\frac{dQ_{\frac{11}{6}}}{d\alpha }=\frac{34\;Q_{\frac{11}{6}}-23\;Q_{\frac{17}{6}}-11\;Q_{\frac{5}{6}}}{6\alpha }.$$

Substituting (A15) into (A14) and collecting terms yields

$$\begin{array}{cc} \frac{dQ_{\frac{5}{6}}}{d\alpha }\; & =\frac{24}{11}\alpha \left(Q_{\frac{17}{6}}-Q_{\frac{11}{6}}\right)+\frac{12}{11}\alpha^{2}\cdot \frac{1}{6\alpha }\left(34\;Q_{\frac{11}{6}}-23\;Q_{\frac{17}{6}}-11\;Q_{\frac{5}{6}}\right)\\ \; & =\left(\frac{24}{11}-\frac{46}{11}\right)\alpha \;Q_{\frac{17}{6}}+\left(-\frac{24}{11}+\frac{68}{11}\right)\alpha \;Q_{\frac{11}{6}}-2\alpha \;Q_{\frac{5}{6}}\\ \; & =-2\alpha \;Q_{\frac{17}{6}}+4\alpha \;Q_{\frac{11}{6}}-2\alpha \;Q_{\frac{5}{6}}\;=\;-2\alpha \left(Q_{\frac{17}{6}}-2\;Q_{\frac{11}{6}}+Q_{\frac{5}{6}}\right)\\ \; & =-2\alpha \;Q_{q}. \end{array}$$

Rearranging immediately gives the identity

(A16) $$\alpha \;Q_{q}\;=\;-\frac{1}{2}\;\frac{dQ_{\frac{5}{6}}}{d\alpha }\;.$$

Using (A16) we can eliminate $dQ_{5/6}/d\alpha$ wherever it appears. Apply it to the differentiated relation (A13) by first expanding the product rule

$$\frac{d}{d\alpha }\;\left(\frac{11}{12\alpha^{2}}\;Q_{\frac{5}{6}}\right)=\frac{11}{12\alpha^{2}}\;\frac{dQ_{\frac{5}{6}}}{d\alpha }-\frac{11}{6\alpha^{3}}\;Q_{\frac{5}{6}},$$

and then substituting $\;\frac{dQ_{5/6}}{d\alpha }=-2\alpha \;Q_{q}\;$ from (A16). The term proportional to $dQ_{5/6}/d\alpha$ produces $-\left(\frac{12}{11}\alpha^{2}+1\right)\frac{11}{6\alpha }\;Q_{q}$, which we move to the left-hand side in (A13). In addition, the combination of $Q_{5/6}$ terms leads to $-11/\left(6\alpha^{3}\right)\;Q_{5/6}$. Altogether this yields the first-order closed equation

(A17) $$\frac{dQ_{q}}{d\alpha }+\left(\frac{12}{11}\alpha^{2}+1\right)\frac{11}{6\alpha }\;Q_{q}=-\frac{11}{6\alpha^{3}}\;Q_{\frac{5}{6}}-\frac{dQ_{\frac{11}{6}}}{d\alpha }.$$

The right-hand side of (A17) solely in terms of $Q_{11/6}$ and $Q_{17/6}$ is

$$-\frac{11}{6\alpha^{3}}\;\left(\frac{12}{11}\alpha^{2}\left(Q_{\frac{17}{6}}-Q_{\frac{11}{6}}\right)\right)-\frac{1}{6\alpha }\;\left(12\alpha^{2}\left(Q_{\frac{17}{6}}-Q_{\frac{11}{6}}\right)-17\;Q_{\frac{11}{6}}\right),$$

by using (A11) and the second equality in (A10). This expression simplifies to

$$-\frac{2}{\alpha }\left(\alpha^{2}+1\right)Q_{\frac{17}{6}}+\frac{2}{\alpha }\left(\alpha^{2}+\frac{29}{12}\right)Q_{\frac{11}{6}},$$

which substituting into the right-hand side of (A17) gives

(A18) $$\frac{dQ_{q}}{d\alpha }+\frac{11}{6\alpha }\left(\frac{12}{11}\alpha^{2}+1\right)Q_{q}=\frac{2}{\alpha }\left(\alpha^{2}+\frac{29}{12}\right)Q_{\frac{11}{6}}-\frac{2}{\alpha }\left(\alpha^{2}+1\right)Q_{\frac{17}{6}}.$$

To obtain a second-order relation for $Q_{q}$, differentiate (A17) with respect to α. On the left-hand side, use the product rule on the coefficient of $Q_{q}$; on the right-hand side, keep $Q_{5/6}$ and $d^{2}Q_{11/6}/d\alpha^{2}$ explicit for the moment. A straightforward calculation gives

(A19) $$\begin{array}{cc} \frac{d^{2}Q_{q}}{d\alpha^{2}} & +\frac{11}{6}\left(\frac{12}{11}\alpha +\frac{1}{\alpha }\right)\frac{dQ_{q}}{d\alpha }+\frac{11}{6}\left(\frac{12}{11}-\frac{3}{\alpha^{2}}\right)Q_{q}=\frac{11}{2\alpha^{4}}\;Q_{\frac{5}{6}}-\frac{d^{2}Q_{\frac{11}{6}}}{d\alpha^{2}}. \end{array}$$

Next eliminate $d^{2}Q_{11/6}/d\alpha^{2}$ by differentiating the second line of (A10), which, by the product rule, gives

$$6\alpha \;\frac{d^{2}Q_{\frac{11}{6}}}{d\alpha^{2}}+6\;\frac{dQ_{\frac{11}{6}}}{d\alpha }=11\;\frac{dQ_{\frac{5}{6}}}{d\alpha }-17\;\frac{dQ_{\frac{11}{6}}}{d\alpha }.$$

Solving this last expression for $d^{2}Q_{11/6}/d\alpha^{2}$ and using (A16), we obtain

$$\frac{d^{2}Q_{\frac{11}{6}}}{d\alpha^{2}}=-\frac{23}{6\alpha }\;\frac{dQ_{\frac{11}{6}}}{d\alpha }-\frac{11}{3}\;Q_{q}.$$

Inserting this into (A19) yields

(A20) $$\begin{array}{c} \frac{d^{2}Q_{q}}{d\alpha^{2}}+\frac{11}{6}\left(\frac{12}{11}\alpha +\frac{1}{\alpha }\right)\frac{dQ_{q}}{d\alpha }-\frac{11}{6}\left(\frac{3}{\alpha^{2}}+\frac{10}{11}\right)Q_{q}=\frac{11}{2\alpha^{4}}\;Q_{\frac{5}{6}}+\frac{23}{6\alpha }\;\frac{dQ_{\frac{11}{6}}}{d\alpha }, \end{array}$$

which is precisely the form used in the subsequent elimination step.

At this point we have three linear equations coupling $Q_{11/6}$, $Q_{17/6}$, and $Q_{q}$: namely (A12), (A18), and the suitable transformation of (A20) (again using (A11) and (A10)). Writing them explicitly as a linear system we obtain

(A21) $$\begin{array}{cc} \left(\frac{12}{11}\alpha^{2}+1\right)Q_{\frac{17}{6}}-\left(\frac{12}{11}\alpha^{2}+2\right)Q_{\frac{11}{6}} & =Q_{q}, \end{array}$$

(A22) $$\begin{array}{cc} -\left(\alpha^{2}+1\right)Q_{\frac{17}{6}}+\left(\alpha^{2}+\frac{29}{12}\right)Q_{\frac{11}{6}} & =\frac{\alpha }{2}\left[\frac{dQ_{q}}{d\alpha }+\frac{11}{6\alpha }\left(\frac{12\alpha^{2}}{11}+1\right)Q_{q}\right], \end{array}$$

(A23) $$\begin{array}{cc} \left(\frac{23\alpha^{2}}{18}+1\right)Q_{\frac{17}{6}}-\left(\frac{23\alpha^{2}}{18}+\frac{23\cdot 17}{18\cdot 12}+1\right)Q_{\frac{11}{6}} & =\frac{\alpha^{2}}{6}\left[\frac{d^{2}Q_{q}}{d\alpha^{2}}+\left(2\alpha +\frac{11}{6\alpha }\right)\frac{dQ_{q}}{d\alpha }\right.\\ \; & \;\left.-\left(\frac{33}{6\alpha^{2}}+\frac{10}{6}\right)Q_{q}\right]. \end{array}$$

This coupled system (A21)–(A23) is solved by standard elimination. Eliminating $Q_{17/6}$ via (A21) from (A22) and (A23), then solving (A22) for $Q_{11/6}$ and substituting into (A23), produces a single second-order homogeneous ODE for $Q_{q}$:

(A24) $$P\left(\alpha \right)\frac{d^{2}Q_{q}}{d\alpha^{2}}+Q\left(\alpha \right)\frac{dQ_{q}}{d\alpha }+R\left(\alpha \right)Q_{q}=0,$$

with polynomial coefficients

$$\begin{array}{cc} P(\alpha ) & =36\alpha^{2}\left(72\alpha^{2}+55\right),\\ Q(\alpha ) & =12\alpha \left(432\alpha^{4}+1554\alpha^{2}+1265\right),\\ R(\alpha ) & =15552\alpha^{4}+23760\alpha^{2}+21505. \end{array}$$

As usual, the first-derivative term can be removed by the exponential integrating factor or Liouville transformation quoted in Equation (A6), which here leads to the dependent variable

$$\psi \left(\alpha \right)\equiv \alpha^{\frac{23}{6}}e^{\alpha^{2}/2}{\left(72\alpha^{2}+55\right)}^{-1/2}\;Q_{q}\left(\alpha \right),$$

satisfying the Schrödinger-type form

(A25) $$\frac{d^{2}\psi }{d\alpha^{2}}-\frac{15552\alpha^{6}+34128\alpha^{4}+58179\alpha^{2}-4180}{3{\left(72\alpha^{2}+55\right)}^{2}}\psi =0,$$

which is equivalent to

$$\frac{d^{2}\psi }{d\alpha^{2}}-\left(\frac{2}{3}+\alpha^{2}+\frac{154}{72\alpha^{2}+55}-\frac{11880}{{\left(72\alpha^{2}+55\right)}^{2}}\right)\psi =0,$$

because

$$\begin{array}{cc} & 3{\left(72s+55\right)}^{2}\;\left[s+\frac{2}{3}+\frac{154}{72s+55}-\frac{11880}{{\left(72s+55\right)}^{2}}\right]\\ & \;=3s{\left(72s+55\right)}^{2}+2{\left(72s+55\right)}^{2}\\ & \;\;+462(72s+55)-35640\\ & \;=15552s^{3}+34128s^{2}+58179s-4180. \end{array}$$

which matches the numerator upon replacing $s=\alpha^{2}$.
